# Parenting Style in Childhood and Mental Health Outcomes of Caregiving in Middle and Later Adulthood

**DOI:** 10.1093/geroni/igab046.2984

**Published:** 2021-12-17

**Authors:** Yujun Liu, M Courtney Hughes, Abby Baumbach

**Affiliations:** 1 Northern Illinois University, Naperville, Illinois, United States; 2 Northern Illinois University, DeKalb, Illinois, United States

## Abstract

Objectives. This study examined the association between remembered parenting style of both mothers and fathers in childhood and mental health outcomes of caregiving in middle and later adulthood. Methods. Data were from the Midlife in the United States (MIDUS) study, a national survey that included 7,108 participants aged 24 to 75 years at baseline. The sample analyzed in the current study included 244 MIDUS participants who had given personal care to their mothers or fathers for one month or more during the last 12 months in the second and third waves. Parenting style variables, which included maternal/paternal affection and maternal/paternal discipline, were from the first wave; mental health outcome variables, which included emotional distress, depressive symptoms, and life satisfaction, were from the second and third waves. Multiple regression and multilevel modeling were applied using R. Results. Maternal affection was negatively associated with emotional distress. Paternal affection was negatively associated with depressive symptoms. The associations between maternal/paternal discipline and mental health outcomes were not significant. Among the caregivers who provided care for parents, those who had mothers with high affection in childhood experienced a lower level of emotional distress, those who had fathers with high affection experienced a lower level of depressive symptoms in middle and later adulthood. Discussion. Our findings have advanced the understanding of the long-term consequences of parenting style in childhood on mental health outcomes among family caregivers in later life. The results have implications in the development of interventions focusing on mental health outcomes among family caregivers.

